# Na^+^, K^+^-ATPase Signaling and Bipolar Disorder

**DOI:** 10.3390/ijms19082314

**Published:** 2018-08-07

**Authors:** David Lichtstein, Asher Ilani, Haim Rosen, Noa Horesh, Shiv Vardan Singh, Nahum Buzaglo, Anastasia Hodes

**Affiliations:** Department of Medical Neurobiology, Institute for Medical Research Israel-Canada, The Hebrew University-Hadassah Medical School, Jerusalem 91120, Israel; asheri@ekmd.huji.ac.il (A.I.); haimr@ekmd.huji.ac.il (H.R.); noa.rosenthal1@mail.huji.ac.il (N.H.); vardanshiva@gmail.com (S.V.S.); buzaglo@gmail.com (N.B.); anastasi.singalevich@mail.huji.ac.il (A.H.)

**Keywords:** bipolar disorder, depression, mania, Na^+^, K^+^-ATPase, cardiac steroids, signaling, ERK, AKT

## Abstract

Bipolar disorder (BD) is a severe and common chronic mental illness characterized by recurrent mood swings between depression and mania. The biological basis of the disease is poorly understood and its treatment is unsatisfactory. Although in past decades the “monoamine hypothesis” has dominated our understanding of both the pathophysiology of depressive disorders and the action of pharmacological treatments, recent studies focus on the involvement of additional neurotransmitters/neuromodulators systems and cellular processes in BD. Here, evidence for the participation of Na^+^, K^+^-ATPase and its endogenous regulators, the endogenous cardiac steroids (ECS), in the etiology of BD is reviewed. Proof for the involvement of brain Na^+^, K^+^-ATPase and ECS in behavior is summarized and it is hypothesized that ECS-Na^+^, K^+^-ATPase-induced activation of intracellular signaling participates in the mechanisms underlying BD. We propose that the activation of ERK, AKT, and NFκB, resulting from ECS-Na^+^, K^+^-ATPase interaction, modifies neuronal activity and neurotransmission which, in turn, participate in the regulation of behavior and BD. These observations suggest Na^+^, K^+^-ATPase-mediated signaling is a potential target for drug development for the treatment of BD.

## 1. Depressive and Bipolar Disorder (BD)

Major depressive disorder, dysthymia, and bipolar disorder (BD), commonly referred to as depressive disorders, are a serious and devastating group of diseases. Affecting some 10% of the population, they pose a significant public health issue. These disorders are manifested by a combination of symptoms that interfere with the ability to work, study, sleep, eat, and enjoy once pleasurable activities. BD is one of the most distinct syndromes in psychiatry and has been described in numerous cultures over the course of history, in a manner suggesting considerable similarity of the syndrome in time and place [1]. BD is characterized by episodes of extreme mood states, mania and depression, interspersed with periods of euthymia. Symptoms of mania include elevated mood, hyperactivity, racing thoughts, insomnia, irritability, and risky behavior. Depression is associated with symptoms such as sad mood, poor self-esteem, lethargy, and anhedonia. The unique phase of the illness is mania. However, depression can be the most prominent phase and the ratio of depression to mania over the course of the disorder is highly variable [2,3]. BD is a frequent disease; depending upon the study, the estimated lifetime prevalence of BD among adults worldwide is 1 to 3% [4]. Family, twin, and adoption studies demonstrate that inherited factors are involved in the pathogenesis of BD [5]. Despite the availability of a broad range of drugs, treatment remains inadequate. Some patients do not respond to treatment and many suffer from frequent relapses [6]. A better understanding of the mechanisms of BD could therefore contribute to the development of targeted therapies and is of the utmost importance.

Despite the devastating impact of BD on millions worldwide, the underlying mechanisms of the etiology and neurobiology of the disease is poorly understood. Historically, the brain systems that receive the greatest attention in neurobiological studies of mood disorders are the monoaminergic neurotransmission, which are distributed extensively throughout the network of limbic, striatal, and prefrontal cortical neuronal circuits that are thought to support the behavioral manifestations of mood disorders [7]. This notion began following the unexpected discovery that reserpine, a drug used for the treatment of hypertension, caused depression in a few patients [8]. Further experimental analysis revealed that reserpine inhibited vesicular monoamine transporters and depleted brain monoamine levels, implicating serotonin and norepinephrine in mood disorder pathobiology [9]. Later, it was shown that administration of monoamine oxidase inhibitors and tricyclic antidepressants altered monoamine neurotransmitter levels and relieved depressive symptoms. These findings gave rise to the hypothesis that monoamine depletion contributes to mood disorder pathology [10], a notion referred to as the monoamine hypothesis. Accordingly, monoamine neuronal reuptake and degradation inhibitors were developed for the treatment of mood disorders. Although this strategy has proved useful in alleviating symptoms, the inhibitors’ slow pace of action (3–5 weeks), extensive side-effects, and poor response in a significant proportion of patients (65–75%) constitute significant limitations [11,12]. Moreover, the fact that monoamine depletion fails to produce depressive symptoms in healthy individuals [13] suggests that additional mechanisms participate in the pathophysiology of mood disorders and BD in particular. To this end, studies in recent years focus on the involvement of additional neurotransmitter/neuromodulators systems and cellular processes in BD. These include alterations in the metabolism and action of cholinergic [14], glutaminergic [15], GABAergic [16], and opioid [17] neurotransmission as well as changes in the activity of proteins located at the post-synaptic densities [18]. In addition, strong evidence showed that mitochondrial function [19,20] and oxidative stress [21] and inflammation [22,23] participate in the etiology of BD: Reduced antioxidant capacity was described in bipolar patients, manifested by decreased levels of glutathione in post-mortem prefrontal cortex samples [24]. Downregulation of a number of antioxidant genes, including Superoxide dismutase (SOD1), was found in BD [25]. Reduced antioxidant capacity leads to the accumulation of Reactive oxygen species (ROS), which, in turn, causes oxidative damage to macromolecules. Indeed, biomarkers indicating oxidative damage were reported in BD patients: higher levels of protein carbonylation and lipid peroxidation [26]. In addition to the permanent changes, several studies found a correlation between manic or depressive mood states and the levels of oxidative damage biomarkers in bipolar individuals [26]. In addition, neurotrophic factors, mainly brain-derived neurotrophic factor (BDNF), are important for neuroplasticity, a process that is impaired in patients suffering from BD [27,28], and Wnt and GSK-3 signaling [29] participate in the etiology of the disease. Despite these findings, none of these directions have led to the development of established anti-depressive or anti-manic drugs and all the available drugs are compounds that modify the monoamine system in the brain [7,12]. For the past 10 years, we and other laboratories presented evidence that the Na^+^, K^+^-ATPase and endogenous cardiac steroids (ECS) are involved in the etiology of BD. Although a complete description of Na^+^, K^+^-ATPase and ECS is beyond the scope of this article, a cursory review of these entities will be presented before focusing on their possible involvement in BD. The reader is referred to the excellent reviews on a more comprehensive presentation on Na^+^, K^+^-ATPase and CS-induced signaling included in this special issue of IJMS.

## 2. Na^+^, K^+^-ATPase

Sodium, potassium-activated adenosine triphosphatase (Na^+^, K^+^-ATPase), an enzyme present in the plasma membrane of most eukaryotic cells, hydrolyzes ATP and uses the free energy to drive the transport of potassium into the cell and sodium out of the cell, against their electrochemical gradients. This pump is the major determinant of the Na^+^ and K^+^ electrochemical gradient. As such, it has an important role in regulating cell volume, plasma membrane electrical potential, as well as cytoplasmic pH and Ca^2+^ levels through the Na^+^/H^+^ and Na^+^/Ca^2+^ exchangers, respectively and in driving a variety of secondary transport processes [30]. Na^+^, K^+^-ATPase is a hetero-oligomer composed of stoichiometric quantities of two major polypeptides: its α and β-subunits. The 100–112 kDa α-subunit is a multi-spanning membrane protein that is responsible for the catalytic and transport properties of the enzyme and contains the binding sites for the cations, ATP, cardiotonic steroids (CS) and a group of regulatory proteins [31]. The β-subunit is a 45–55 kDa type II glycoprotein that transverses the membrane once and is part of the functional core of the pump and is required for its trafficking to the plasma membrane [32]. A third protein, FXYD, named after a shared PFxYD motif in the N terminal extracellular part of the single transmembrane protein, is associated with Na^+^, K^+^-ATPase and modulates ion transport [33]. There are four genes encoding the α-subunits α1, α2, α3, and α4, four genes encoding the four β isoforms β1, β2, β3, and β4, and seven genes encoding the seven FXYD isoforms. The α, β and FXYD-isoforms exhibit a species-, tissue-, and cell-specific pattern of expression. Their distribution has been extensively studied and reviewed [30].

The α1 subunit is essentially omnipresent at the tissue and cellular levels. The α2 isoform is predominantly expressed in muscle (heart and skeletal) and brain (in astrocytes and glia cells) [34]. The α3 isoform is mainly expressed in the brain, ovaries, and white blood cells [35]. In the brain this isoform is mainly localized in neuronal projections [36] and to some extent in dendritic spines [37]. All three β subunits, which affect the kinetic properties of the pump, reducing the apparent potassium affinity and raising the extracellular sodium affinity, are found in the brain. Of the seven FXYD proteins, at least five (FXYD1 (phospholemman), FXYD2 (gamma-subunit of Na^+^, K^+^-ATPase), FXYD3 (Mat-8), FXYD4 (CHIF), and FXYD7), are auxiliary subunits of Na^+^, K^+^-ATPase and regulate pump activity in a tissue- and isoform-specific way [30,33,38].

## 3. Na^+^, K^+^-ATPase and Behavior 

Numerous studies have shown that mutations in the Na^+^, K^+^-ATPase α isoform elicit behavioral changes. Moseley and colleagues showed that α1 heterozygous mice exhibit an increased locomotor response to AMPH, whereas α2 heterozygous mice show reduced locomotor activity and increased anxiety-related behavior [39,40]. The α3 heterozygous mice displayed spatial learning and memory deficits, increased locomotor activity, and an increased locomotor response to methamphetamine. Schaefer and colleagues found that the α2-ouabain resistance mutation (α2R/R) caused decreased locomotor activity, impaired learning, and increased responsiveness to methamphetamine [41]. The heterozygous mice for the loss-of-function disease-mutation G301R in the α2 isoform (α_2_^+/G301R^) shows hypo-locomotion in female mice and a stronger response to aversive acoustic stimuli of both males and females, compared with WT mice [42]. Mice harboring a heterozygous hot spot disease mutation, D801Y (α3^+/D801Y^) in the α3 isoform exhibited hyper-locomotion relative to WT mice and increased sensitivity to chemically-induced epileptic seizures [43]. And finally, Myshkin mice carrying an inactivating mutation in the α3 subunit display deficits in social behavior [44], circadian disruptions [45] as well as increased exploratory locomotion and sensitivity to AMPH [46]. Cumulatively, these studies strongly support the notion that Na^+^, K^+^-ATPase activity is involved in determining behavior.

## 4. Cardiac Steroids (CS) and Endogenous CS (ECS)

Cardiac steroids, which include cardenolides (such as ouabain and digoxin), and bufadienolides (such as bufalin and marinobufagenin), have been used for centuries, and are used today to treat cardiac failure, arrhythmias, and other maladies in Western and Eastern medicine [47,48,49,50]. In the past few decades, compounds similar or identical to CS were identified in mammalian tissues. These include ouabain [51], digoxin [52], and several bufadienolide-like compounds such as 19-norbufalin [53], 3β-hydroxy 14α 20:21-bufenolide [54], proscillaridin A [55], marinubufagenin [56], and telocinobufagin [57]. The most studied ECS is the ouabain-like steroid. The presence of endogenous ouabain was demonstrated in numerous studies showing the presence of a compound that interacts with specific and sensitive anti-ouabain antibodies and which was consequently purified and identified according to mass spectrum analysis [58]. Although this steroid was found in human plasma and urine more than 25 years ago, its exact structure is still under debate. Some claim that the endogenous ouabain is indistinguishable from the plant steroid [51,59], others maintain that the mass spectrum data relating to the endogenous steroid do not support this conclusion [60,61,62]. Clearly, additional analytical studies are required to solve this dispute. The biosynthetic pathway for these steroids in mammalian tissue has not been established. However, numerous studies support the notion that endogenous ouabain is synthesized in and released from the adrenal gland and hypothalamus [63,64]. Furthermore, results of experiments with a radioactive tracer chase support the notion that cholesterol is the substrate for the synthesis of cardenolides and that cholesterol side-chain cleavage and 3β hydroxylation are the first reactions in this process [65,66]. On the other hand, it was recently demonstrated in human trophoblast and rat adrenocortical cells that the biosynthesis of marinobufagenin from cholesterol occurs via a novel acidic bile acid pathway [56]. The lack of detailed information on the biosynthesis of the ECS impedes the acceptance of these steroids as hormones. Clearly, studies based on substrate utilization, inhibitors, and tracer methods, in combination with chromatographic and mass spectrum analyses, are crucial. Despite this limitation, many consider the ECS a hormone family involved in numerous physiological processes and pathological states, including salt homeostasis and regulation of blood pressure, cell growth, and differentiation and behavior [59,67,68,69,70,71,72].

## 5. Na^+^, K^+^-ATPase-Induced Intracellular Signaling

It is now accepted that in addition to its main transport function, Na^+^, K^+^-ATPase also acts as a signal transducer. The pioneering observation that the addition of low concentrations of ouabain to cultured neonatal cardiac myocytes or A7r5 smooth muscle cells rapidly activates Src [73] set the ground for intense and versatile research into the signaling processes of CS-Na^+^, K^+^-ATPase interactions. For almost 20 years, research on the molecular basis of the CS-induced signaling, unequivocally led by Dr. Zijian Xie and his colleagues, has been conducted in many laboratories. These hundreds of studies have established that the interaction of CS with Na^+^, K^+^-ATPase is directly responsible for the activation of signal transduction cascades in cardiac myocytes, renal epithelial cells, neuronal, and several other cell types. The signaling activates Src, phopholipase C, MAPK, Akt, and reactive oxygen species, slows Ca^2+^ oscillation, and consequent NFκB activation [74,75]. It is also well recognized that Na^+^, K^+^-ATPase-mediated signaling is involved in many physiological processes, including cell growth, differentiation, inflammation, muscle contractility, kidney function, and behavior (as described in detail in IJMS in this Journal). In most, if not all, studies Na^+^, K^+^-ATPase-mediated signaling is manifested following the addition of CS. Hence, the so-called Na^+^, K^+^-ATPase-mediated signaling is actually CS-Na^+^, K^+^-ATPase-mediated signaling and strengthens the versatile roles of the ECS. Importantly, the activation of the intracellular signaling reactions by CS-Na^+^, K^+^-ATPase interactions occurs at cardenolide and bufadienolide concentrations (nM and sub-nM) similar to those found in the human circulation [59,67,68,69,71,76].

## 6. Na^+^, K^+^-ATPase and ECS in BD

Genetic, molecular, behavioral, and pharmacological studies in the past decade provided strong evidence for the involvement of the Na^+^, K^+^-ATPase/ECS system in BD:

1. An allelic association between BD and a Na^+^, K^+^-ATPase α subunit gene (ATP1A3) has been reported [77]. The significant association with BD of six single SNPs in the three genes of the Na^+^, K^+^-ATPase α isoforms, suggests that this enzyme plays a role in the etiology of the disease [78]. It was also shown that a genetic dysfunction of the neuron-specific Na^+^, K^+^-ATPase α3 isoform (Myshkin mice) induces manic-like behavior [79].
2. BD has been consistently associated with abnormalities in Na^+^, K^+^-ATPase activity in erythrocytes [80,81]. Meta-analysis of erythrocyte Na^+^, K^+^-ATPase activity in bipolar illness showed a significant mood-state-related decrease in the enzyme’s activity in both manic and BD patients [82]. Furthermore, Na^+^, K^+^-ATPase density was significantly lower in BD patients than in major depressed and schizophrenic patients [83]. In addition, a reduction in brain Na^+^, K^+^-ATPase α1 isoform expression was found in mice treated with the mood stabilizer lithium [83].
3. The plasma levels of endogenous CS were significantly reduced in manic individuals, compared with those in normal controls [84,85]. The levels of these compounds were increased in the parietal cortex of post mortem samples from BD patients, vs schizophrenic, major depressed, and normal individuals [86].
4. Numerous studies have demonstrated that intracerebroventricular (i.c.v.) injection of ouabain induces hyperactive behavior in rats [87,88,89]. Actually, some studies refer to an ouabain-induced increase in activity as an animal model for mania [89,90,91]. Indeed, CS-induced hyperlocomotion is reduced following the administration of lithium or valporic acid, common mood stabilizers used in the treatment of bipolar disorder [92].
5. The i.c.v. administration of highly specific and sensitive anti-ouabain antibodies, which lower brain ECS, resulted in anti-depressive effects, as measured in the forced swimming test in normal rats [86] as well as in the Flinder Sensitive Line (FSL) of genetically depressed rats [93]. In addition, administration of anti-ouabain antibodies also elicited anti-depressive effects in lipopolysaccharide-treated rats, another animal model of depression [86]. Furthermore, this treatment caused significant changes in catecholamine metabolism in the hippocampus and ventral tegmentum, two areas know to be associated with mood disorders [93].
6. Administration of amphetamine (AMPH), a potent central nervous system stimulant, to BALB/c and black Swiss mice, resulted in a marked increase in locomotor activity, accompanied by a threefold increase in brain ECS [94]. The reduction in brain ECS by i.c.v. administration of anti-ouabain antibodies prevented the AMPH-induced hyperactivity and the increase in brain ECS levels [94].
7. AMPH caused oxidative stress in the hippocampus and frontal cortex, manifested by an increase in SOD and a decrease in CAT and GPx activity, and a reduction in NPSH and an increase in TBARS levels. The reduced brain ECS activity following i.c.v. administration of anti-ouabain antibodies protected against these AMPH-induced effects [95].

## 7. Na^+^, K^+^-ATPase Signaling and BD

**Figure 1:**
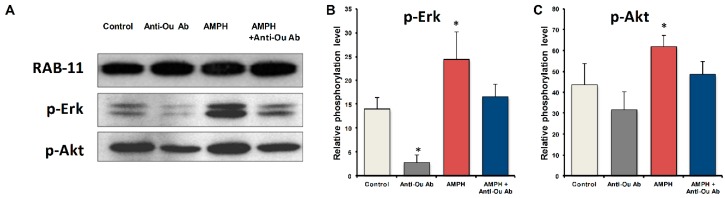
Effect of amphetamine and anti-ouabain antibodies on ERK and Akt phosphorylation levels in the frontal cortex. Male BALB/c mice were administered saline (10 mL/kg IP) and nonspecific IgG (1 µg/kg ICV) (Control, *n* = 10), saline and anti-ouabain antibodies (1 µg/kg i.c.v.) (Anti-Ou Ab, *n* = 10) or AMPH (5 mg/kg IP) and IgG (AMPH, *n* = 10) or AMPH and anti-ouabain antibodies (AMPH + Anti-Ou Ab, *n* = 10). The mice were sacrificed and the protein levels were determined by Western blot analysis. The values are presented as the mean ± SE (error bars). * *p* < 0.05. (This figure was adapted with permission from Hodes A, Rosen H, Deutsch J, Lifschytz T, Einat H., and Lichtstein D. Endogenous cardiac steroids in animal models of mania. Bipolar Disorder. 2016 Aug; 18(5):451–9).

As described above and in detail in this issue, by interacting with Na^+^, K^+^-ATPase, CS activate several intracellular signaling pathways, including ERK and Akt phosphorylation. Administration of ouabain in the lateral brain ventricle in rats resulted in mania-like hyperactivity, affording this experimental perturbation an animal model for mania [87,92,96]. In addition, ouabain administration induced a dose-dependent increase in Akt phosphorylation in the frontal cortex, striatum, and hippocampus [97]. Phosphorylation of GSK-3β (Ser9), FOXO1 (Ser256), and eNOS (Ser1177), all downstream molecules of Akt, was also increased in a dose-dependent manner within the same brain regions [98]. It was also well documented that the in vivo ouabain treatment stimulated dose-dependently the MEK1/2-ERK1/2-p90RSK pathway [99]. These findings suggested that the activation of these signaling pathways may underline the behavioral effects induced by ouabain. We recently examined the effect of the CNS stimulant amphetamine (AMPH) and the reduction in brain ECS resulting from i.c.v. injection of specific anti-ouabain antibodies on behavior and ERK and Akt phosphorylation in the mouse frontal cortex [94]. The results showed a reduction in AMPH-induced hyperactivity [94], implicating the ECS in behavior. Furthermore, we have shown that anti-ouabain antibody administration causes reduction in basal ERK phosphorylation in the mouse frontal cortex (Figure 1). In agreement with previous studies [100,101], AMPH induced a 75% and 41% increase in p-ERK and p-Akt levels, respectively, in the frontal cortex (Figure 1). The administration of anti-ouabain antibodies significantly reduced the AMPH-induced increase in the phosphorylation levels of the two proteins (Figure 1). These results suggest that the manic-like phase is characterized by activation of the ERK and Akt signaling pathways in the frontal cortex, which is attenuated by a reduction in ECS levels. It is tempting to propose that the alterations in ERK and Akt phosphorylation caused by changes in ECS are mediated by their interactions with Na^+^, K^+^-ATPase. Such a sequence of events was proposed for the CS-induced effects on stimulation of cell viability [102], increased heart contractility [103,104], and kidney development [105].

**Figure 2:**
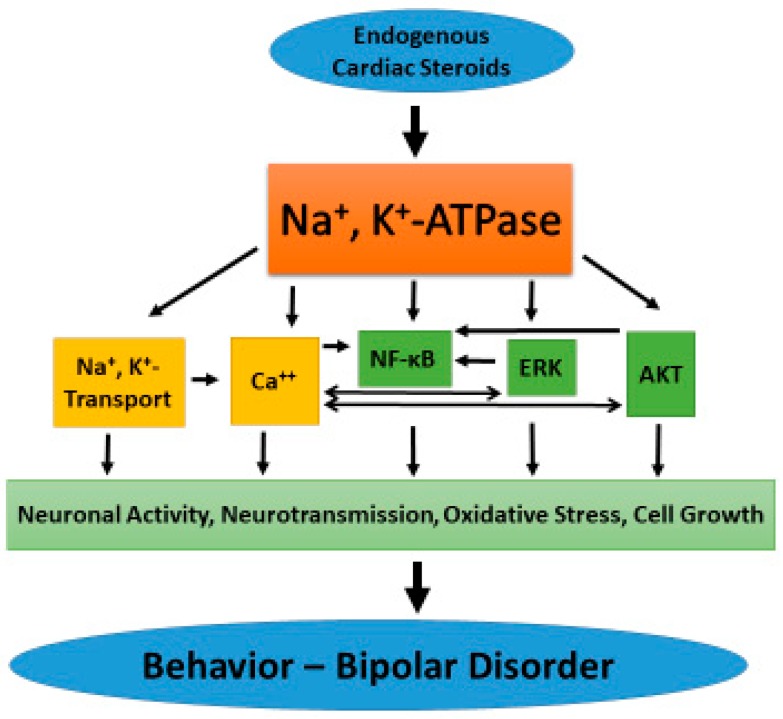
Schematic representation of the link between ECS, Na^+^, K^+^-ATPase and BD. See text for details.

The possible link between Na^+^, K^+^-ATPase activity and signaling and BD is depicted in Figure 2. Human or animal behavior, like all brain functions, is underlined by neuronal electrical activity and synaptic transmission. Na^+^ and K^+^ gradients across the plasma membrane, established by the ion transporting activity of Na^+^, K^+^-ATPase, are the main determinants of the resting membrane potential, directly influencing neuronal activity [106]. Synaptic transmission is also affected by the ionic gradients and is influenced by the inhibition of Na^+^, K^+^-ATPase activity by CS [107,108,109]. In addition, as described above, there is strong evidence showing that the interaction of CS with Na^+^, K^+^-ATPase induces the activation of intracellular signaling cascades, including Ca^2+^ oscillation and ERK, AKT, and NFκB activation. It is well established that alterations in these intracellular signaling have profound effects on synaptic transmission and plasticity. This was documented repeatedly for ERK [110,111,112], AKT [113,114,115], and NFκB [116,117,118] activations.

## 8. Prospect and Future Directions

BD is a heterogeneous condition with a myriad symptoms varying in manifestation; dysregulation of numerous biochemical pathways has been suggested to be involved in its pathogenesis. Research in Na^+^, K^+^-ATPase-induced signaling is evolving. The goal of this overview was not to draw definitive conclusions about Na^+^, K^+^-ATPase signaling in BD but to summarize the current knowledge, and to discuss limitations and shortcomings in the existing research. The emerging literature provides exciting initial evidence suggesting that alterations in Na^+^, K^+^-ATPase signaling is involved in BD. However, additional work is necessary in order to establish a causal relationship between the two. The uncovering of the metabolism and physiological role of ECS in the brain is the fundamental need. Furthermore, pharmacological experiments evaluating the effects of ERK, AKT, and NFκB inhibitors on behavior and examination of the consequence of alterations in ECS metabolism on Na^+^, K^+^-ATPase signaling may provide important information on the issue. A deeper and clearer understanding of the Na^+^, K^+^-ATPase-induced signaling cascades will establish a better understanding of the complex mechanisms underlying the pathophysiology of BD and may lead to new venues for the development of novel targets for the treatment of this disease.

## 9. Search Strategy

This review was based on search in the PUBMED data base for the key words “bipolar disorder” or “depression” or “mania” with “Na^+^, K^+^-ATPase”, “ouabain”, “cardiac steroids”, “intracellular signaling”, “ERK”, “AKT”, and “NFκB”. No language or time constraints were applied. The lists of references were searched manually to find additional articles
